# Selenium nanoparticles based on *Amphipterygium glaucum* extract with antibacterial, antioxidant, and plant biostimulant properties

**DOI:** 10.1186/s12951-023-02027-6

**Published:** 2023-08-03

**Authors:** Jorge J. O. Garza-García, José A. Hernández-Díaz, Janet M. León-Morales, Gilberto Velázquez-Juárez, Adalberto Zamudio-Ojeda, Jenny Arratia-Quijada, Oscar K. Reyes-Maldonado, Julio C. López-Velázquez, Soledad García-Morales

**Affiliations:** 1 Plant Biotechnology, Centro de Investigación y Asistencia en Tecnología y Diseño del Estado de Jalisco, Camino Arenero 1227, 45019 Zapopan, Mexico; 2 Coordinación Académica Región Altiplano Oeste, Universidad Autónoma de San Luis Potosí, Carretera Salinas-Santo Domingo 200, 78600 Salinas de Hidalgo, Mexico; 3 Centro Universitario de Ciencias Exactas e Ingenierías, Universidad de Guadalajara, Boulevard Gral. Marcelino García Barragán 1421, 44430 Guadalajara, Mexico; 4 Departamento de Ciencias Biomédicas, Centro Universitario de Tonalá, Universidad de Guadalajara, Av. Nuevo Periférico Oriente 555, 45425 Tonalá, Mexico; 5 Plant Biotechnology, CONAHCYT-Centro de Investigación y Asistencia en Tecnología y Diseño del Estado de Jalisco, Camino Arenero 1227, 45019 Zapopan, Mexico

**Keywords:** Antimicrobial activity, Antioxidant compounds, Green synthesis, Plant growth improvement

## Abstract

**Background:**

In recent years, crop production has expanded due to the variety of commercially available species. This increase in production has led to global competition and the search for biostimulant products that improve crop quality and yield. At the same time, agricultural products that protect against diseases caused by phytopathogenic microorganisms are needed. Thus, the green synthesis of selenium nanoparticles (SeNPs) is a proposal for achieving these needs. In this research, SeNPs were synthesized from methanolic extract of *Amphipterygium glaucum* leaves, and chemically and biologically characterized.

**Results:**

The characterization of SeNPs was conducted by ultraviolet–visible spectrophotometry (UV–Vis), scanning electron microscopy (SEM), electron microscopy transmission (TEM), Dynamic Light Scattering (DLS), energy dispersion X-ray spectroscopy (EDX), and infrared spectrophotometry (FTIR) techniques. SeNPs with an average size of 40–60 nm and spherical and needle-shaped morphologies were obtained. The antibacterial activity of SeNPs against *Serratia marcescens*, *Enterobacter cloacae*, and *Alcaligenes faecalis* was evaluated. The results indicate that the methanolic extracts of *A*. *glaucum* and SeNPs presented a high antioxidant activity. The biostimulant effect of SeNPs (10, 20, 50, and 100 µM) was evaluated in vinca (*Catharanthus roseus*), and calendula (*Calendula officinalis*) plants under greenhouse conditions, and they improved growth parameters such as the height, the fresh and dry weight of roots, stems, and leaves; and the number of flowers of vinca and calendula.

**Conclusions:**

The antibacterial, antioxidant, and biostimulant properties of SeNPs synthesized from *A. glaucum* extract demonstrated in this study support their use as a promising tool in crop production.

**Graphical Abstract:**

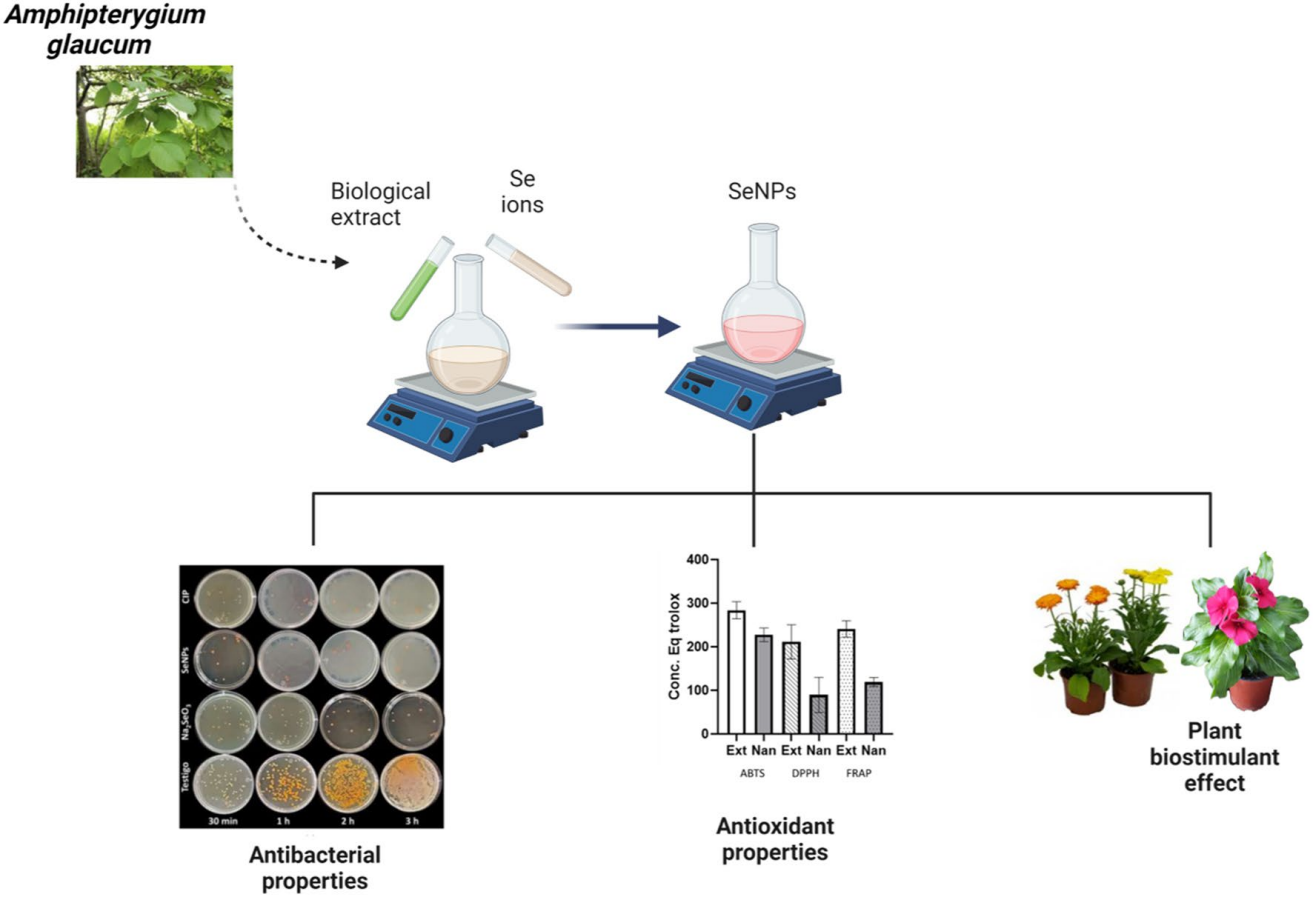

**Supplementary Information:**

The online version contains supplementary material available at 10.1186/s12951-023-02027-6.

## Background

Agricultural production increasingly demands more inputs focused on improving crop production, maintaining plant health, increasing the post-harvest life of products, and generating market-added value of plant foods by enhancing their nutritional properties and organoleptic characteristics [51]. To address this demand, chemical fertilizers, and pesticides have been developed. However, their excessive use can cause direct or indirect contamination of air, water, soil, and the ecosystem, causing health risks to people [60]. In view of this, innovative products based on nanotechnology have begun to be generated that represents a potential alternative and that, with their use, could exponentially reduce the toxicity induced by commercial products. At the same time, agricultural production increases with the formulation of nanopesticides, nanofertilizers, and nanobiostimulants [27]; improving the diagnosis, control of plant diseases and post-harvest management of plant species of commercial interest [30].

The commonly used elements for the synthesis of NPs are silver (Ag) and gold (Au). However, the resulting NPs could have restricted application due to their toxicity at specific concentrations [74]. Although their effects also depend on the method of synthesis. AgNPs and AuNPs obtained by biological synthesis have shown high potential to be used in crop production due to their stability and lower toxicity to plants [22, 44].

In addition, other chemical elements such as selenium (Se) has been proposed to obtain NPs (SeNPs). Selenium is commonly found in nature and is essential in the diet of humans, mammals, and many other life forms [68]. Although Se is not considered essential for plants, it is classified as a beneficial element that enhances crop growth, development, and quality [18]. This element has a dual biological activity on plants since low concentrations promote growth and development, and high concentrations produce toxic effects. The beneficial effect of selenium has been observed in some plant species, such as bell pepper (*Capsicum annuum*) and tomato (*Solanum lycopersicum*) plants, thought increased plant height, growth, and dry weight of leaves and roots [54]. For *Calendula officinalis* seedlings, the beneficial was shown with an increased plant height, leaf area, leaf number, and leaf dry weight [24]. Selenium source also influences plant development, in particular, SeNPs caused higher vegetative development, yield, and nutritional quality of cowpea (*Vigna unguiculata*) seeds [13]. In chili and chicory (*Cichorium intybus*) plants, treatment with SeNPs increased fresh biomass almost twofold [1, 63]. Depending on the plant species, bulk Se was more toxic than SeNPs at concentrations above 10 µM or 1 mg/L. SeNPs can also intervene in synthesizing proteins, carbohydrates, and vitamins, increasing the chlorophyll content in leaves and improving photosynthesis [13, 31]. In addition, SeNPs could control the attack of phytopathogens due to their antimicrobial activity [59] and reduce stress in plants by acting as an antioxidant agent [20].

Among the methodologies employed for synthesizing SeNPs, green methods have been proposed as an alternative to the use of toxic reducing chemical agents. The formation of SeNPs, by green synthesis, requires the addition of bioactive compounds of biological interest known as secondary metabolites, which are found in organisms such as fungi, bacteria, and plants. The use of plant extracts for NPs synthesis is considered a more stable and easier to execute method due to the elimination of cell maintenance of the microorganisms employed for synthesis [40]. Also, plant extracts act as reducing/stabilizing agents that favor the production of NPs through the main groups of secondary metabolites contained in plant extracts, such as terpenoids and flavonoids [22, 40]. These plant secondary metabolites are characterized by diverse biological properties such as antimicrobial, anti-inflammatory, antioxidant, and antiviral properties [25]. Thus, during the synthesis of NPs, some properties, such as antioxidant properties found in plant extracts, can be potentiated [15].

*Amphipterygium glaucum* is a dioecious tree and belongs to the Anacardiaceae family. There are few reports on the biological properties of *A. glaucum*, Gómez-Cansino et al. [19] reported the potent antibacterial activity of both bark stem and leaf extract against *Mycobacterium tuberculosis* and moderate anti-HIV-RT (human immunodeficiency virus -reverse transcriptase) activity. These properties found in *A. glaucum* can be potentiated through their application for green methods NPs synthesized.

*Calendula officinalis* belongs to the Asteraceae family. It is a medicinal herbaceous plant characterized by the phytochemical composition of its flowers and leaves, highlighting phenolic compounds, flavonoids, saponins, carotenoids, triterpenoids, and essential oils [3]. This species is known for its antiviral, anti-inflammatory, anticancer, antidiabetic, antioxidant, and antimicrobial properties [58].

This study used a methanolic extract of *A. glaucum* leaves (CHE1) to synthesize and stabilize SeNPs. The SeNPs obtained were characterized by spectroscopy techniques such as UV–vis and FTIR, the latter to recognize the functional groups present in the extract and in the SeNPs. The presence of Se in the synthesized NPs was verified by EDX spectroscopy. SEM and TEM electron microscopy were used to analyze the size and morphology of the SeNPs. The antibacterial activity of SeNPs was evaluated in three bacteria of foodborne and phytopathological importance (*S. marcescens*, *E. cloacae*, and *A. faecalis*) using colony-forming unit counting methodology. The antioxidant activity of the CHE1 extract and SeNPs was also determined, as well as the concentration of reducing sugars, free amino acids, polyphenolic compounds, and flavonoids. Finally, the biostimulant effect of SeNPs on the plant development, root growth, and flowering of *Calendula officinalis* and *Catharanthus roseus* was evaluated.

## Materials and methods

### Processing of extracts

Leaves of *A. glaucum*, taxonomically identified and deposited at the National Laboratory of Plant Identification and Characterization (LaniVeg) of the Institute of Botany (IBUG) of the University of Guadalajara, Mexico under the registration number SIST-TRA-2018-7, were used. The plant material was collected in the town of La Huerta, Jalisco, Mexico (19°29′24.2′′N 105°02′33.9′′W). The leaves were selected and cleaned to remove contaminating particles to obtain the extracts. Then, the plant material was frozen at − 80 °C (Forma 900, Thermo Fisher, Waltham, USA) and freeze-dried (Free zone, Labconco, Kansas City, USA) for 5 d. Subsequently, the dried plant tissue was ground in an industrial mill (MF10BS1, Ika Werke, Wilmington, USA) and extracted three times by maceration with methanol at a 1:10 (w/v) ratio for 24 h. Methanol was evaporated in a rotary evaporator (R-100, Buchi, Flawil, Switzerland) at 40 °C under reduced pressure. The methanolic extract of *A. glaucum* leaves (CHE1) was stored at − 80 °C and then lyophilized to obtain a powder. The dried extract was held at 4 °C to preserve the nature and properties of the compounds present in the extract.

### SeNP synthesis

For the synthesis of SeNPs, 80 µL of CHE1 extract (50 mg/mL) was added to Na_2_SeO_3_ solution (Sigma-Aldrich, St. Louis, USA) at the final concentration of 10 mM. The synthesis reaction was kept under constant stirring at 1200 rpm for 40 min at 40 °C. Then, the reaction was achieved in the dark with constant stirring for 24 h at room temperature. The colloidal solution was stored at 4 °C for further analysis and use.

### SeNP characterization

The synthesis of SeNPs was confirmed by observing absorption peak maxima between 200 and 500 nm in a spectrometer (Genesis 10S UV–Vis, Thermo Scientific, Carlsbad, USA). The contribution of possible functional groups of *A. glaucum* leaf extracts to the synthesis of SeNPs was evaluated by FTIR analysis (Nicolet iS 5, Thermo Scientific, Waltham, USA) in the 4000-400 cm^−1^ region. Morphological characterization of SeNPs was performed by SEM (MIRA 3 LMU, TESCAN, Brno, Czech Republic) at 20 kV and TEM (JSM-1010, JEOL, Tokyo, Japan) at 80 kV. To determine the size distribution of SeNPs dispersed in the aqueous medium, the DLS technique was performed using a Zetasizer (Nano-ZS90, Malvern Instrument, United Kingdom). The elemental composition of the SeNPs was determined by energy dispersive X-ray spectroscopy (EDS) using a microprobe for surface microanalysis (Quantax EDS, Bruker, Billerica, Germany).

### Antibacterial activity of SeNPs

The antimicrobial activity was evaluated on *Serratia marcescens, Enterobacter cloacae*, and *Alcaligenes faecalis* strains, which were identified at the molecular level previously by Hernández-Díaz et al. [26]. The microorganisms were grown in nutrient broth (BD Bioxon, Mexico) for *S. marcescens* and *E. cloacae* and LB broth (Sigma-Aldrich, USA) for *A. faecalis.* The strains were grown at 37 °C for 18–24 h in a natural convection incubator (Incucell IC 55, MMM Group, Munich, Germany). Subsequently, an inoculum was prepared with peptonized water (Difco, Detroit, USA) and adjusted to a concentration of 0.5 on the McFarland scale (1.5 × 10^8^ CFU/mL), a densitometer (Densimat, Biomérieux, Lyon, France) was used. Bacterial inocula were treated with a solution of SeNPs (5 mM), Na_2_SeO_3_ salt (5 mM), and ciprofloxacin (CIP, 1 mg/mL) for 30, 60, 120, and 180 min at 37 °C. After the incubation time with the treatment had elapsed, seven 1/10 serial dilutions were performed. From the last dilution, using the surface spreading technique, a 100 µL aliquot of each treatment was plated in duplicate Petri dishes with nutrient agar. The untreated inoculum was used as a negative control. The inoculated plates were incubated at 37 °C for 24 h. Finally, CFU from each plate was counted, and the results of three independent assays were reported as CFU/mL.

### Antioxidant activity determination

The antioxidant activity of the CHE1 extract and SeNPs was determined by DPPH, ABTS^+^, and FRAP techniques, as described by Hernández-Díaz et al. [26]. For the DPPH method, absorbance was measured at 515 nm in a spectrometer (Multiskan Sky High, Thermo Scientific, Vantaa, Finland). For the ABTS technique, the reaction was monitored for 30 min, with readings every 3 min at 754 nm. While for the FRAP assay, the reaction mixture was incubated for 30 min and read at 595 nm.

In all three assays, a Trolox standard curve was performed in concentrations between 50 and 400 µM. The results were expressed in Trolox equivalents in µM (TE).

### Determination of bioactive compounds

Reducing sugars, free amino acids, flavonoids, and total polyphenols were determined according to Alonso et al. [6]. A brief description of the method is given below.

The determination of reducing sugars in CHE1 extract and SeNPs was carried out as follows: a working solution of 1% (w/v) di-nitrosalicylic acid (DNS) with 36% (w/v) sodium potassium tartrate dissolved in 0.5 M NaOH was prepared. In a glass test tube, 500 µL of the working solution was mixed with 500 µL of the sample. Then, the glass test tube was heated in water at 95 °C for 5 min. The sample was allowed to cool, 4 mL of water was added, and the solution was read in triplicate at 575 nm. A standard curve was prepared using glucose in concentrations between 0.2 and 1 g/L.

Free amino acids were determined using 2 mL of each sample placed in test tubes, followed by 1 mL of ninhydrin reagent (Sigma, St Louis, USA). The mixture was heated for 10 min at 95 °C. Subsequently, 4 mL of 95% (w/v) ethanol was added. Finally, the absorbance reading was taken at 570 nm. Glycine was used for the standard curve in a concentration range between 5 and 50 µM.

Flavonoids were quantified by the aluminum trichloride (AlCl_3_) method, following the methodology described by Alonso et al. [6]. Results were expressed as quercetin equivalents per mL of sample.

Polyphenolic compounds were determined with the Folin–Ciocalteu reagent with the methodology reported by Alonso et al. [6]. Results are presented as gallic acid equivalents in µg/mL (GAE).

### Biostimulant effect of SeNPs in ornamental species

Seeds of vinca (*C. roseus*) cv. Tattoo Black Cherry (Ball, Chicago, USA) and calendula (*C. officinalis*) cv. Costa Orange (Ball, Chicago, USA) were used. Seeds were sown in 72-cavity polyethylene germination trays with commercial peat moss substrate. Subsequently, the trays were incubated at 25 °C with photoperiods of 16 h light and 8 h dark, and the substrate was maintained with watering periods of three times per week. The 24-d-old seedlings were transplanted into polyethylene pots (1.5 L) with a mixture of peat moss and perlite under greenhouse conditions (average temperature of 24 °C, average relative humidity of 59.2%, and average solar radiation of 537 W/m^2^). The plants were irrigated with tap water four times per week until the start of the treatments. At 25 days after transplanting, different concentrations of SeNPs (0, 10, 20, 50, or 100 µM) were foliar sprayed for 10 weeks with an application every seven days. Two weeks after the last application of the treatments, all growth parameters as the root length and volume, fresh and dry weight of roots, leaves, stems, and flowers, the number of leaves, branches, and flowers, the diameter of stems and flowers, plant height were determined following the methodology reported in Hernández-Díaz et al. [24] and Saldaña-Sánchez et al. [54]. The plants were grown for 102 days from transplant to harvest.

The chlorophylls *a* and *b*, total chlorophyll, and carotenoids were quantified as previously reported [54]. Photosynthetic efficiency was measured with the portable chlorophyll fluorometer (OS1p Fluorometer, Opti-Sciences, Hudson, USA), following the instructions in the manual for the Y(II) protocol.

### Statistical analysis

For the antibacterial activity of SeNPs, a two-way analysis of variance (ANOVA) was conducted in SAS 9.1 statistical software (SAS Institute, Cary, USA); also, the difference between the mean values was determined using Duncan's multiple range test with a significance level of *p* < 0.05.

For antioxidant and biostimulant activities, data were processed by one-way analysis of variance (ANOVA) using Duncan's multiple range test, for which the statistical package Stat graphics Centurion XV (Stat graphics, USA) was used with a significance level of *p* < 0.05.

## Results and discussion

### Synthesis and characterization of SeNPs

SeNPs were obtained from the reduction of Na_2_SeO_3_ semimetallic ions with the methanolic extract of *A. glaucum* to induce the formation of nucleation centers, resulting in SeNPs forming. In addition, in this reaction, the extract stabilized the SeNPs. Similar results were reported where *A. adstringens* extracts can act as reducing and stabilizing agents during the synthesis of silver NPs [50].

**Fig. 1:**
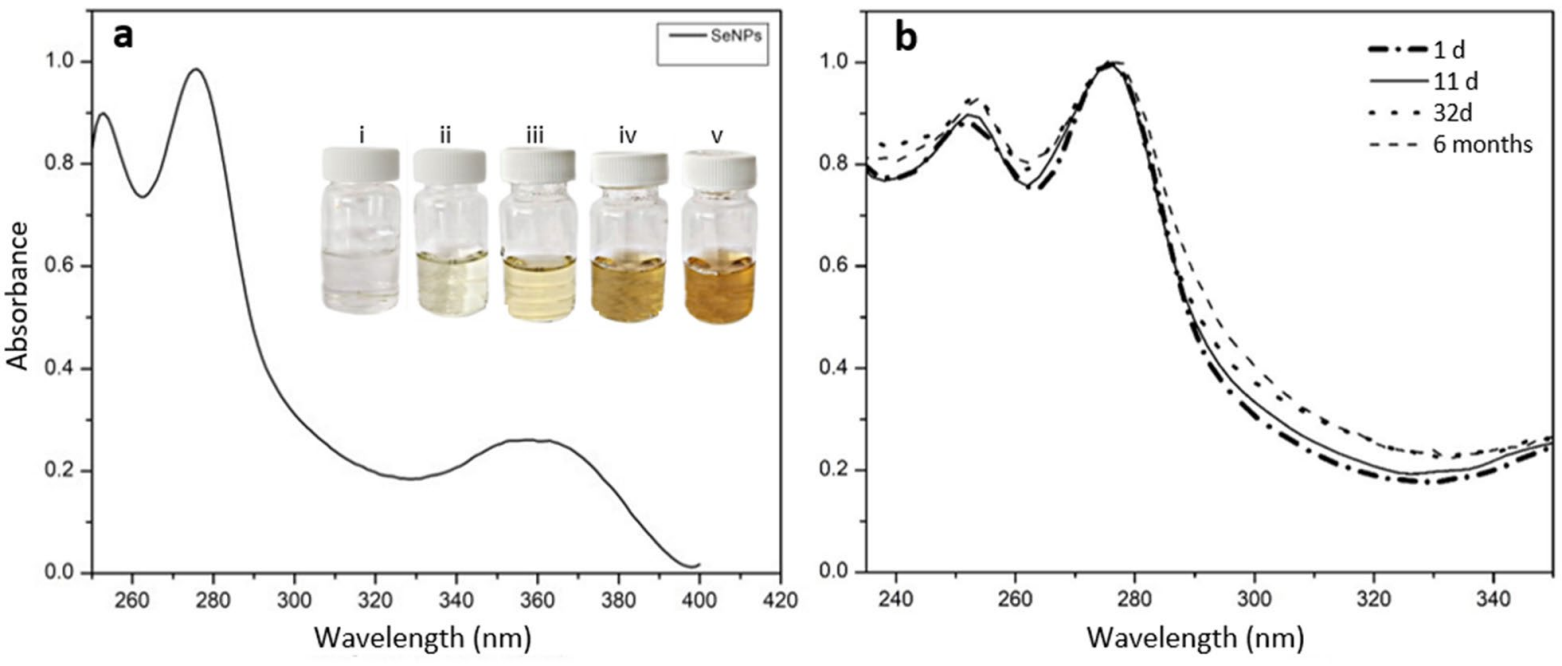
UV–Vis spectra of SeNPs. **a** Typical absorption peak for SeNPs and bioreduction during SeNPs synthesis mediated by methanolic extract of *A. glaucum* leaves at 0 (i), 10 (ii), 20 (iii), 30 min (iv), and 24 h (v) after the reaction initiation. **b** The stability of the SeNPs is shown by the absorption spectra measured at different times

In all experiments, adding the CHE1 extract over the aqueous Na_2_SeO_3_ solution led to the bioreduction of the solution. It was monitored by the color change from light green to reddish-yellow due to the excitation of surface plasmon vibrations in the SeNPs [69]. In this work, the reduction of Se ions present in the Na_2_SeO_3_ aqueous solution during the reaction with the CHE1 extract was observed by UV–Vis spectroscopy in a range from 200 to 400 nm, and the maximum absorption was obtained at 275 nm. The CHE1 extract exposed to Na_2_SeO_3_ ions presented an absorption of approximately 275 nm (Fig. 1a), which corresponds to the surface plasmon resonance (SPR) for SeNPs reported at 270 nm by Gunti et al. [20] through the use of *Emblica officinalis* and at 265 nm with the use of *C. officinalis* extracts [26].

The stability of SeNPs was recorded by UV–Vis spectra from an aqueous solution of Na_2_SeO_3_–CHE1 extract from 24 h to 6 months. The plasmon resonance band of the surface plasmon of Se started at approximately 275 nm after 24 h. The high surface energy of NPs makes them far from the equilibrium state and extremely unstable, which could cause changes in their properties [20]. The absorption maximum peak at 275 nm remains constant with the passage of days, demonstrating high stability of the solution until the last evaluation performed 6 months after the SeNPs syntheses (Fig. 1b). This long stability effect could be related to the stabilizing nature of the CHE1 extract protecting from agglomeration and extending its stability.

**Fig. 2:**
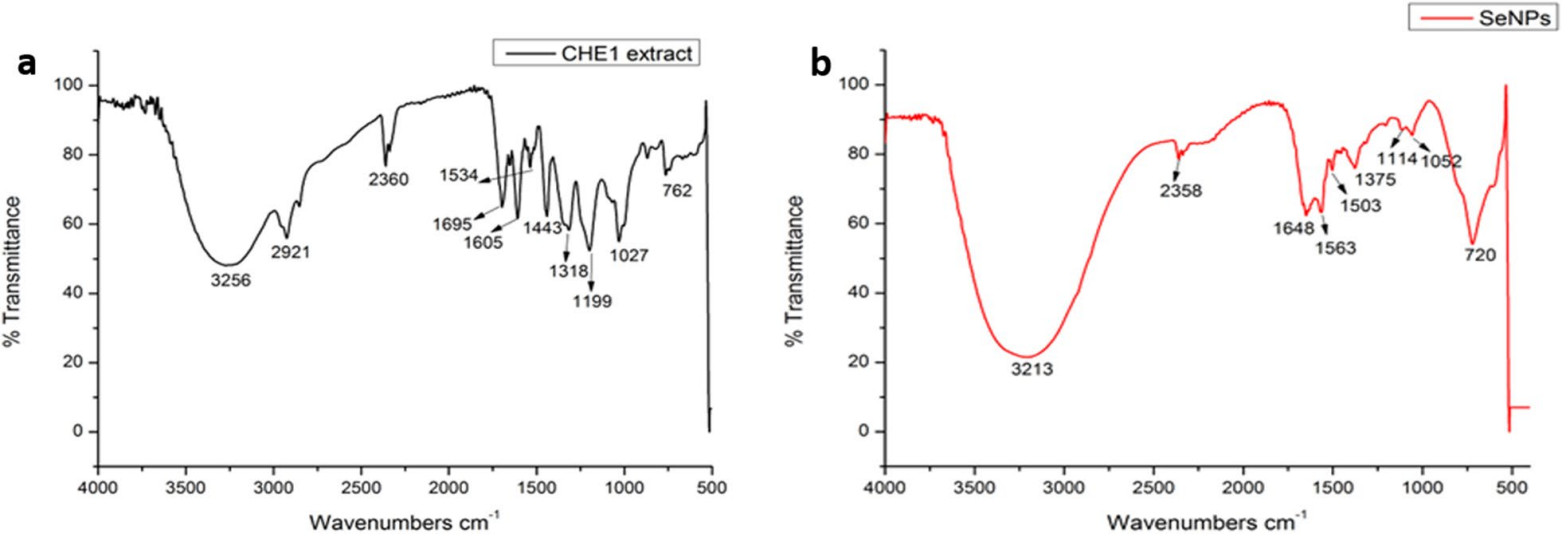
FTIR spectroscopy analysis. **a** FTIR spectra of the extract of *A. glaucum*. **b** FTIR spectra of the SeNPs

FTIR spectral measurements were performed to identify the functional groups of biomolecules present in the extracts potentially involved in the reduction of Na_2_SeO_3_ ions to the formation of SeNPs and their stabilization (Fig. 2a, b). Representative peaks of the functional groups of CHE1 extract and their interaction with Na_2_SeO_3_ were found. According to the analyzed data, the ability of CHE1 extracts to reduce semimetallic ions to SeNPs and to act as a stabilizing agent for SeNPs by binding to the surface of SeNPs is suggested. According to the results presented in the FTIR spectra, absorption bands at 3256 cm^−1^ responsible for the stretching vibrations (O–H) of the extract were identified, suggesting the presence of this group as responsible for the reduction of Se to SeNPs (Fig. 2a). For the case of SeNPs, this band was shifted to 3213 cm^−1^ (Fig. 2b), indicating a strong interaction between the -OH groups of the extract and Se by hydrogen bridging [39]. The asymmetric stretching vibration for CO_2_ is observed at 2360 cm^−1^ in both spectra as a noise signal.

Many scientific reports have established that plant extracts contain various phytocompounds, including phenolics and tannins. The reaction of NP formation could initiate from reducing molecules such as polyphenols containing hydrolyzable tannins, which release glucose, gallic acid, and ellagic acid [38, 57, 72]. The band at 2921 was assigned to chain stretching mainly of polysaccharides and functional groups such as carboxylic acid (Fig. 2a), which biomolecules are possibly responsible for stabilizing SeNPs [19]. Likewise, the CHE1 extract exhibited bands at 1443 cm^−1^, corresponding to the C–C stretching of aromatic compounds, and 1318 cm^−1^ (Fig. 2a), attributable to the stretching of the OH groups of phenols [28]. The bands at 1027 and 1114 cm^−1^ indicate the presence of carboxylic acids [45]. The bands at 762 and 720 cm^−1^ could be related to metal–oxygen bond stretching vibrations (Fig. 2b), which could indicate the binding of SeNPs to the –OH groups present in the extract, giving rise to Se–O coordination bonds [20]. These phytochemical compounds exert reducing power to carry out the synthesis of NPs. At the same time, the anionic forms of these acids are transformed into their corresponding quinonic form, yielding electrons, which favors the reduction of the metal ions to their zero valences [8].

Bands were found at 1695, 1605, and 1199 cm^−1^ corresponding to stretching and vibrational bending of C=C, NH_2_, COOH, CH_2_, and C=O (Fig. 2a), indicating the presence of reducing groups in the CHE1 extract responsible for the reduction of SeNPs [47]. This is observed in Fig. 2b as the light shifted to 1648, 1563, and 1114 cm^−1^. Therefore, according to the theory of hard and soft acids and bases, the hydroxyl groups ^−^OH present in the hydrolyzed phenolic compounds of tannins would serve as complex ligands, while the carbonyl groups –CO= would serve as soft ligands. Upon contact of the soft metal ions with the complex ligand, the reduction of the metal to its zero valences occurs. Subsequently, the –CO= group of the weak ligand in the oxidized polyphenols binds with the NPs and stabilizes through electrostatic interactions [72].

On the other hand, a slightly asymmetric absorption band of the SeNPs with indications of an additional weaker component was found at approximately 255 nm (Fig. 1a). This additional band indicates the formation of stable aggregates of SeNPs in solution or polymorphic SeNPs. These results agree with Gunti et al. [20] using *E. officinalis* fruit extracts to reduce Na_2_SeO_3_.

**Fig. 3:**
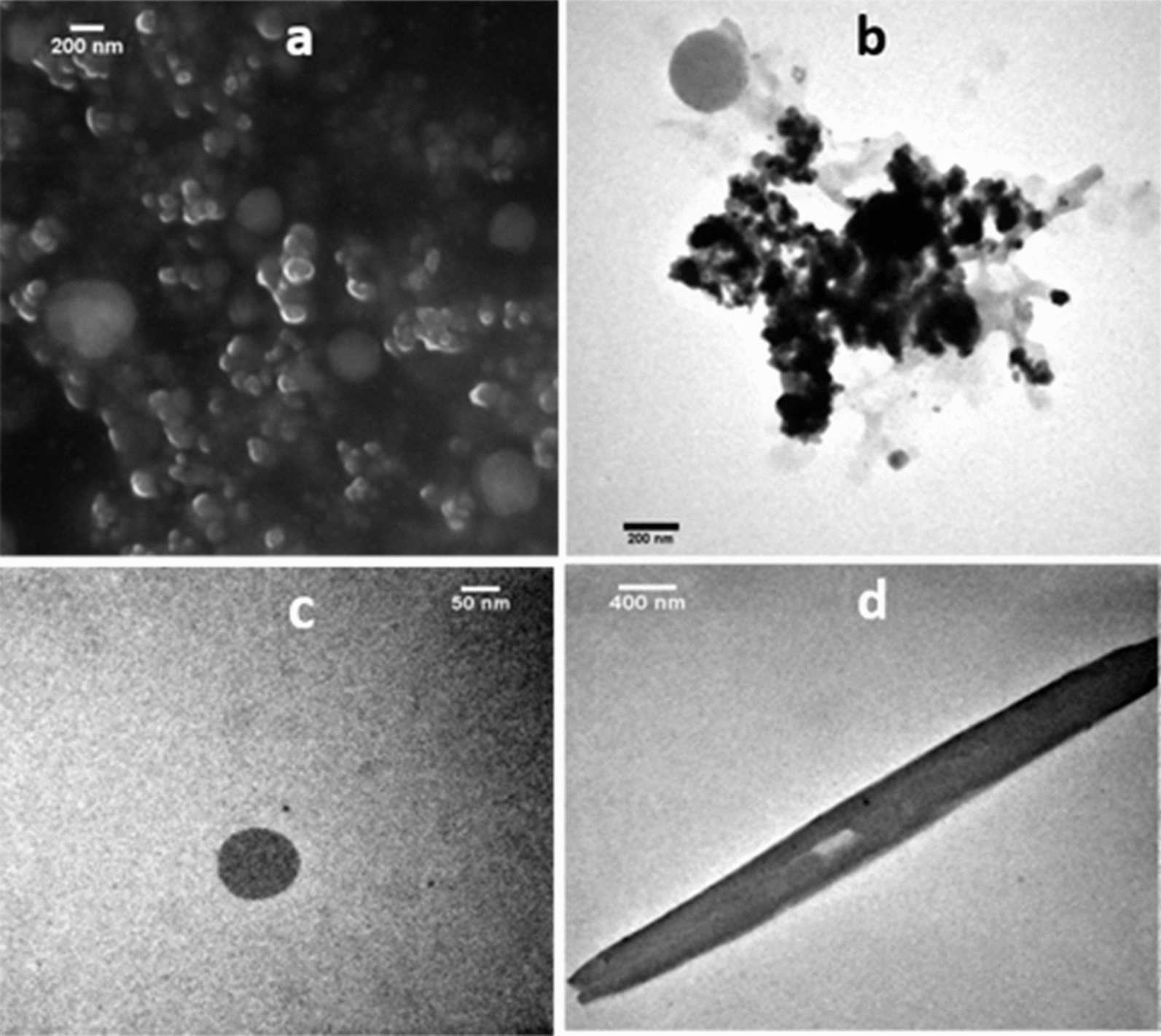
Characterization of SeNPs. **a** SEM micrograph of SeNPs with a magnification of 60 kx. TEM micrographs of SeNPs at **b** 100 kx. **c** 300 kx. **d** 50 kx

The size and morphology of SeNPs were analyzed through SEM (20 kV) using backscattered electrons. The SeNPs obtained from CHE1 extracts presented a spherical/oval morphology with an approximate size of 100 nm (Fig. 3a). Similar results have been reported using extracts of *E. officinalis* [20] and *C. officinalis* [26]. In both cases, the use of secondary metabolites obtained from the extraction allowed the reduction/stabilization of SeNPs with similar morphologies.

To corroborate the size and morphology of the biosynthesized SeNPs, TEM (90 kV) was used. For this case, the micrographs obtained showed a uniform distribution and confirmed a spherical morphology for the SeNPs (Fig. 3b). The presence of SeNPs in the form of nanobars was also appreciated (Fig. 3d), and this could explain the appearance of the second absorption peak (255 nm) found in the UV–Vis spectra reported above due to the presence of two excitation modes. The synthesis of nanoparticles through green methodologies allows obtaining of nanoparticles of amorphous nature as described in other research works using plant extracts such as *C. annuum*, *Spirulina* polysaccharide, and *Emblica officinalis* [56, 20, 73].

**Fig. 4:**
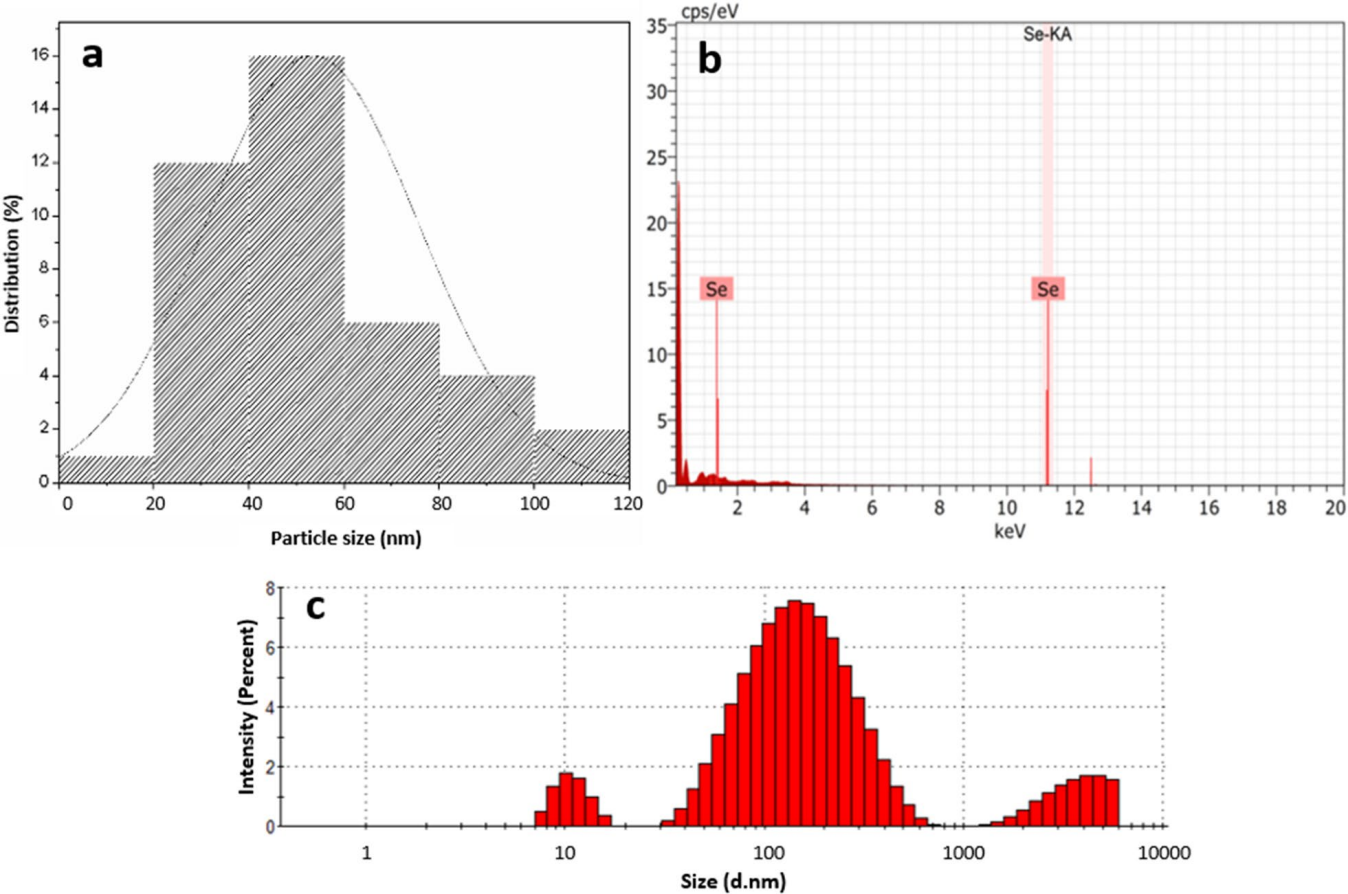
Size and analysis of SeNPs. **a** Size distribution of SeNPs by TEM (90 kV). **b** Elemental analysis of SeNPs. **c** Size determination of SeNPs (d.nm) by dynamic light scattering (DLS)

The size distribution of the SeNPs was determined from the diameters found through the micrographs obtained by TEM. From these results, an average size of 40–60 nm in diameter was established, confirming at the same time a spherical morphology (Fig. 4a). This result agrees with that presented by Rodríguez-Luis et al. [50]. The AgNPs obtained with *A. adstringens* extracts displayed particle sizes and morphologies similar to those obtained in this work with *A. glaucum* extracts. Similarly, using this same species achieved the synthesis of AuNPs with sizes below 50 nm with different morphologies, with spherical AuNPs having the highest proportion [45].

The presence of SeNPs in the analyzed sample was confirmed by EDS analysis (Fig. 4b), identifying characteristic Se absorption peaks at 1.37 keV (SeLα peak) and 11.22 keV (SeKα peak). Similar to what was previously reported during the analysis of the SeNPs [9].

The size distribution of SeNPs was corroborated by DLS, a lower intensity peak representing the presence of nanoparticles smaller than 20 nm was found, and a variety of sizes from 40 to 400 nm was also observed; whereas the most abundant NPs were centered at 142 nm (Fig. 4c). Which may be due to the hydrodynamic coating of water molecules around the SeNPs and the needle-like morphology detected by TEM (Fig. 3d). In this sense, DLS as a method to determine the size of NPs is unreliable for obtaining quantitative data, as this technique does not take into account sample-specific limitations [16]. Previously, it has been mentioned that obtaining the size distribution of SeNPs by DLS could be considered insufficient to know the exact size of SeNPs [35]. This could be due to the fact that the hydrodynamic measurements of SeNPs size obtained by DLS were performed considering all the molecules present in the liquid phase. Moreover, it is necessary to consider that the DLS method is mainly based on the intensity of light scattered by SeNPs in aqueous solutions, where larger NPs will significantly overlap compared to smaller NPs, resulting in a larger average hydrodynamic size [16].

### Antibacterial activity of SeNPs

**Fig. 5:**
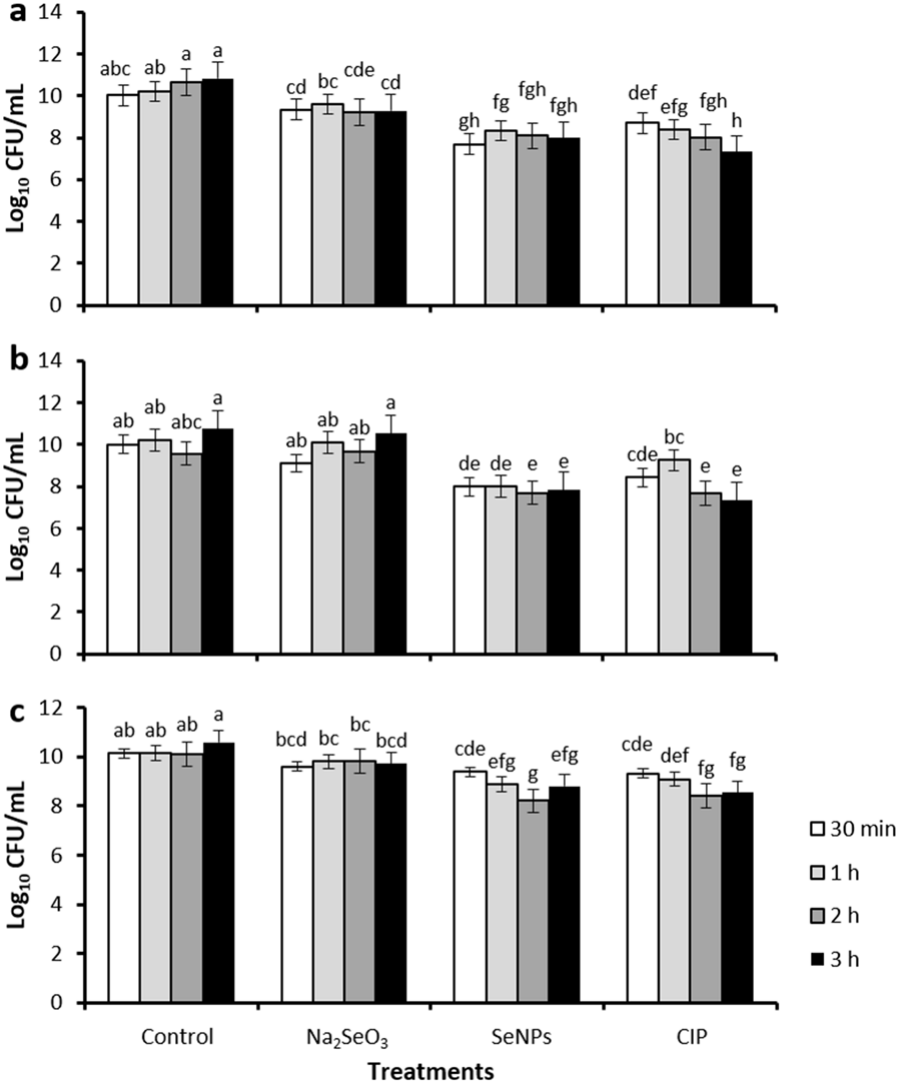
Antibacterial activity of SeNPs. **a** Evaluating antibacterial activity under different treatments and incubation times against *S. marcescens*. **b** *A. faecalis.* **c** *E. cloacae*. *CIP* Ciprofloxacin. Mean values ± SE. According to Duncan's test (α = 0.05), different letters indicate statistically significant differences, p < 0.0001

The antibacterial activity was evaluated over three foodborne bacterial strains (*S. marcescens*, *A. faecalis*, and *E. cloacae*). The results show an inhibitory effect on bacterial growth with the use of SeNPs similar to that obtained by CIP, except for the first 30 min, where a superior antibacterial effect of SeNPs was observed versus *S. marcescens* but not other strains tested (Fig. 5a). Similar results have been observed by Hernández-Díaz et al. [26], who reported the antibacterial effect of SeNPs biosynthesized with ascorbic acid and calendula flower extracts (1.3 mg/mL), showing total inhibitory activity against *S. marcescens* after 2 h. Some of the proposed antibacterial mechanisms of action for SeNPs include depolarization and disruption of the bacterial membrane and inhibition of biofilm formation [26]. These results are relevant because *S. marcescens* is a clinically important bacterium cataloged within the plant pathogens group. It causes yellow vine disease in plants such as watermelon, pumpkin, melon, and squash [62].

The use of CIP presented an inhibitory effect on the growth of the *A. faecalis* strain after 30 min of incubation (Fig. 5a); this effect is expected for an antibiotic. The interesting result was the fact that the SeNPs had a greater inhibition on the growth of this bacterial strain. The bacterial growth showed colonies with small sizes, suggesting a modification of the reproductive and metabolic capacity due to stress in the presence of SeNPs. The inhibitory result by Na_2_SeO_3_ was not comparable with that obtained by SeNPs (Fig. 5a, b), where the antibacterial activity was highest with SeNPs. Although, a decrease in the number of colonies of *S. marcescens* was observed with Na_2_SeO_3_ after 2 and 3 h (Fig. 5a). These results agree with investigations where the antibacterial activity of Na_2_SeO_3_ against *Klebsiella planticola*,* Escherichia coli, Bacillus subtilis*, and *Staphylococcus aureus* was established [4].

On the other hand, SeNPs followed a behavior comparable to CIP at all measured times, except for 1 h, where SeNPs showed a higher CFU inhibition effect than CIP for *A. faecalis*. In this regard, the concentration used for SeNPs (0.8 mg/mL) was lower than that used for CIP (1 mg/mL), which highlights the antibacterial effect achieved by SeNPs. Similarly, Hegerova et al. [23] mentioned that using SeNPs against this bacterium produces growth inhibition. This effect could be partly due to the inhibitory activity on biofilm formation that anacardic acids have demonstrated in extracts of the genus *Amphipterygium* [7].

Regarding a possible mechanism by which these compounds act, anacardic acids are inhibitors of bacterial histidine protein kinase in two-component regulatory systems [7]. These proteins are involved in the regulation of virulence factor expression through quorum sensing (communication mechanism between unicellular organisms) [7]. Therefore, *A. glaucum* extract for forming SeNPs could possess some of these compounds and act similarly through SeNPs.

For *A. faecalis*, using Na_2_SeO_3_ did not present an inhibitory effect on the CFU count from 30 min. For this treatment, the results for all times evaluated were statistically equal to those of the control (Fig. 5b). This could indicate an adaptation of the bacteria to Na_2_SeO_3_ similar to what occurred with *S. marcescens*.

The antibacterial activity of SeNPs was evaluated against *E. cloacae,* and the application of SeNPs presented a similar behavior for CIP. However, the results obtained showed that this bacterial strain presented higher resistance to both treatments, resulting in statistical similarities for all treatment times evaluated (Fig. 5c). Recently, *E. cloacae* has been identified as a plant pathogen and associated with onion decay and the yellowing of papaya [17]. In addition, it has been reported to be pathogenic in orchids [65]. The main symptoms caused on plants consist of leaf wilting, dark brown discoloration, and leaf defoliation in the final stage of infection [17].

Likewise, the use of Na_2_SeO_3_ followed the same trend found for both *S. marcescens* and *A. faecalis* since a statistically comparable number of CFU was observed with the control from 30 min. However, after 3 h, an inhibitory effect of Na_2_SeO_3_ was found concerning the control (Fig. 5c). In agreement with this behavior, the ability of *E. cloacae* to reduce Na_2_SeO_3_ to SeNPs has been reported. This could indicate a possible mechanism of eliminating the toxic agent by this microorganism to reduce the antibacterial activity of SeNPs [26].

The antibacterial effect of SeNPs on Gram-bacteria was comparable to that of the CIP antibiotic and, in some cases, better (Fig. 5). Although, the dose used in this study (0.8 mg/mL) was higher than those reported in other studies (16–256 μg/mL) with human pathogenic bacteria, both Gram+ (*S. aureus*, *S. mutans*, *L. monocytogenes*) and Gram− (*P. aeruginosa*, *E. coli*) [2, 70], as well as plant pathogens such as *Clavibacter michiganensis* subsp. *sepedonicus* [37].These differences shown by the bacterial species studied can be explained by the fact that the high concentration of selenium nanoparticles possibly led the bacteria to metabolize them to selenite, hydrogen selenide, or selenophosphate, and finally to incorporate selenium into selenoproteins, thus maintaining bacterial growth and resistance [29], but the molecular mechanism is poorly understood.

### Antioxidant activity and bioactive compounds of SeNPs

**Fig. 6:**
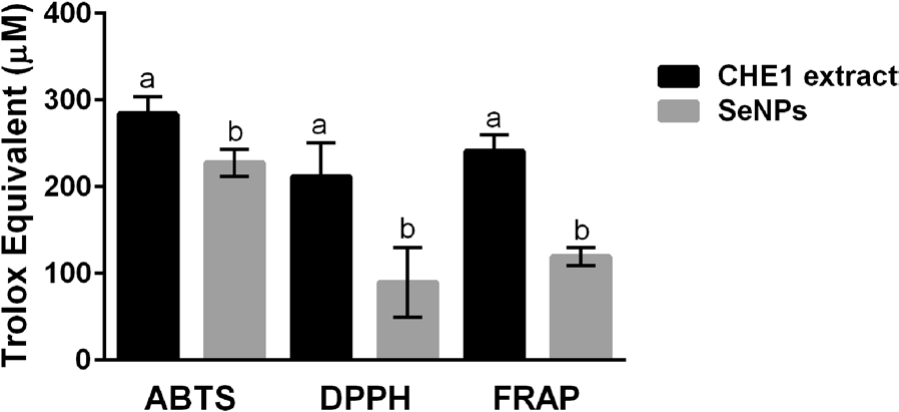
Antioxidant activity of plant extract (CHE1) and SeNPs determined by ABTS (2,2-azino-bis-3-ethylbenzothiazoline-6-sulfonic acid), DPPH (2,2-diphenyl-1-picrylhydrazyl), and FRAP (Ferric reducing antioxidant power) assays. Mean values ± SE. According to Duncan's test (α = 0.05), different letters denote statistically significant differences, p < 0.0001

For this work, the antioxidant activity of both SeNPs and CHE1 extract was evaluated by ABTS, DPPH, and FRAP techniques (Fig. 6). In the determinations performed by the ABTS method; the CHE1 sample presented an antioxidant activity (in Trolox equivalent, TE μM) of 283.8 ± 19.6. At the same time, the value obtained for the SeNPs was 227.5 ± 15.5 TE. This difference in the activity values between the nanoparticles and the extract is an indicator that some molecules with antioxidant potential were consumed during the synthesis of SeNPs to perform the reduction of the nanoparticles.

The determinations performed with the methodologies of DPPH and FRAP maintained the same trend in the decrease of activity, observing the following results for the DPPH test 211.3 ± 38.8 and 89.4 ± 39.8 TE for the CHE1 extract and SeNPs, respectively. Finally, the results evaluated with the FRAP method were 240.7 ± 18.5 and 119.3 ± 9.9 TE for CHE1 and SeNPs, respectively. The SeNPs and CHE1 samples showed higher activity in reducing the ABTS radical than the other evaluated methodologies. The results obtained are consistent with those reported by other studies [26, 48] where the antioxidant capacity detected by ABTS is significantly higher than that observed by DPPH; this phenomenon is a function of the type of antioxidant present, the mechanism of action and the solvents used.

The measurements of bioactive compounds performed in this work show significant differences when comparing the content of the compounds in the plant extract versus the range of these compounds at the end of the nanoparticle synthesis. The concentration of reducing sugars, free amino acids, flavonoids, and polyphenols was higher in the extract than in the SeNPs; this indicates that some of these molecules could mediate as reducing agents in the reaction. Notice that total polyphenols content decreased by two times while total flavonoids decreased almost three times in the SeNPs concerning the CHE1 extract (Table 1). The antioxidant potential of biosynthesized SeNPs could directly depend on the properties of the phytochemical compounds present on their surface and the size of the NPs, as smaller diameters have shown higher antioxidant activity [41]. Similar data have been found in investigations of AgNPs biosynthesized with extracts of *Plantago lanceolata*. A higher extract antioxidant activity than the NPs is shown due to the high content of compounds such as reducing sugars, glycosides, anthraquinone, and tannins [57].

**Table 1:** Total content of chemical compounds in the extract of *A. glaucum* and SeNPs

Treatment	Reducing sugars (mg/L)	Free amino acids (µM)	Flavonoids (QE μg/mL)	Polyphenols (GAE μg/mL)
Plant extract	344.20 ± 11.7a	56.33 ± 4.2a	192.40 ± 12.2a	164.10 ± 1.6a
SeNPs	222.50 ± 12.8b	34.15 ± 2.3b	69.16 ± 9.6b	82.02 ± 3.1b

Mean values ± SE. Different letters in each column denote statistically significant differences according to Duncan's test (α = 0.05), p < 0.0001

On the other hand, there are multiple reports of biosynthesized SeNPs with good antioxidant activity, such as those described by Kumar et al. [33]. They obtained SeNPs from a free extract of *Geobacillus* cells. Likewise, Pyrzynska and Sentkowska [46] obtained SeNPs through a green synthesis mediated by *Aloe vera* with excellent antioxidant activity, evaluated by DPPH and FRAP techniques, and a clear trend of increase in antioxidant activity was observed, with increasing concentration of SeNPs and *A. vera* extract.

Polyphenol content tends to decrease to close to 50% of the content of the CHE1 extract compared with SeNPs (Table 1). In this way, the antioxidant activity and the extracted polyphenols may be responsible for the reduction of Se during the SeNPs synthesis, as a high content of total polyphenols was reported in an analysis of the tissue composition of *A. glaucum* [53]. For their part, Liang et al. [38] previously reported the participation of polyphenols in the synthesis of SeNPs.

Similarly, there are reports that some free amino acids such as arginine, cysteine, histidine, lysine, methionine, tryptophan, and tyrosine have antioxidant activity. These amino acids could be related to the extract’s antioxidant activity and the SeNPs' functionalization with the phytochemical compounds on their surface [71].

Concerning the other families of biomolecules (Table 1), the presence of proteins was not detectable in none of the samples; in addition to demonstrating the relationship of these molecules with the antioxidant capacity of both the extract and the SeNPs [32], the interaction of these molecules with the formation of SeNPs was observed [36].

### Biostimulant effect of SeNPs on the growth of vinca plants

This research evaluated the application of SeNPs as a biostimulant agent in vinca plants under greenhouse conditions. The use of NPs in agriculture is constantly growing because these materials can be applied in small quantities, either by foliar spraying or in nutrient solutions, to improve crop yield and quality [30]. This biostimulation process aims to improve nutrient absorption and increase tolerance to abiotic stress, generating better plant quality. Likewise, NPs can enhance vital plant processes’ performance under specific concentrations, translating into high yields and better crop quality [49].

SeNPs at different concentrations induced significant changes in parameters such as height and the number of leaves and flowers of vinca plants (Additional file 1: Fig. S1). For example, plants treated with the concentrations of 10 and 20 μM presented a greater plant height (6% in both cases) to the control. (Table 2). The same trend was found by El-Batal et al. [12], who reported that foliar application of SeNPs (0.5 mg/L) significantly increased potato plant height. This effect could be due to a possible contribution of SeNPs to the accumulation of phytohormones such as cytokines (CK) and gibberellins (GA), which are plant hormones responsible for regulating plant growth and development [5, 18]. An example of this effect was reported by El Lateef Gharib et al. [13], who indicated that SeNPs had a promoting effect on the levels of the growth hormones GA, CK, and indole-acetic acid (AUX) in cowpea (*Vigna unguiculata*) leaves. In turn, SeNPs reduced the content of abscisic acid (ABA), which could indicate a mechanism of action for SeNPs over phytohormones levels [13].

**Table 2:** Biostimulant activity of SeNPs on vinca plants growth and photosynthetic pigment content

SeNPs (µM)	RM	FWL (g)	FWS (g)	DWS (g)	NF	FWF (g)	DWF (g)	Chl*a *(µg g^−1^ FW)	Chl*b* (µg g^−1^ FW)	Chl*t* (µg g^−1^ FW)	CR (ng g^−1^ FW)	PE
Control	13.6 ± 0.8c	38.2 ± 1.2 b	16.2 ± 0.3 b	3.8 ± 0.1c	29.7 ± 0.7b	6.1 ± 0.3c	0.7 ± 0.1c	2.4 ± 0.2bc	0.9 ± 0.1b	3.5 ± 0.2b	0.15 ± 0.1c	0.60 ± 0.1a
10 µM	16.0 ± 0.4ab	46.2 ± 1.4 a	19.1 ± 0.9a	4.2 ± 0.1a	43.3 ± 1.9a	9.0 ± 0.3ab	1.1 ± 0.1ab	2.2 ± 0.2c	0.8 ± 0.1b	3.1 ± 0.3b	0.17 ± 0.1ab	0.60 ± 0.1a
20 µM	17.2 ± 0.3a	43.8 ± 1.6 a	19.3 ± 0.6a	4.0 ± 0.1ab	42.4 ± 1.3a	8.4 ± 0.5b	0.9 ± 0.1bc	2.3 ± 0.1c	0.8 ± 0.1b	3.1 ± 0.1b	0.19 ± 0.1ab	0.60 ± 0.1a
50 µM	15.3 ± 0.4b	46.6 ± 2.0a	20.2 ± 0.6a	4.3 ± 0.1a	47.8 ± 3.3a	10.5 ± 0.8a	1.1 ± 0.1a	3.5 ± 0.2a	1.1 ± 0.1a	4.7 ± 0.2a	0.20 ± 0.1a	0.58 ± 0.1a
100 µM	15.2 ± 0.4b	45.6 ± 1.2a	18.5 ± 0.7a	4.0 ± 0.1ab	43.2 ± 2.7a	9.6 ± 1.0ab	1.0 ± 0.1ab	2.9 ± 0.1ab	0.9 ± 0.1a	3.9 ± 0.2b	0.21 ± 0.1a	0.56 ± 0.1a

Mean values ± DE. Different letters in each column denote statistically significant differences according to Duncan's test (α = 0.05), p < 0.0001

RM, Ramifications; FWL, Fresh weight leaves; FWS, Fresh weight stem; DWS, Dry weight stem; NF, Number of flowers; FWF, Fresh weight flowers; DWF, Dry weight flowers; Chl*a*, Chlorophyll *a*; Chl*b*, Chlorophyll *b*; Chl*t*, Total chlorophylls; CR, Carotenoids; PE, Photosynthetic efficiency

**Fig. 7:**
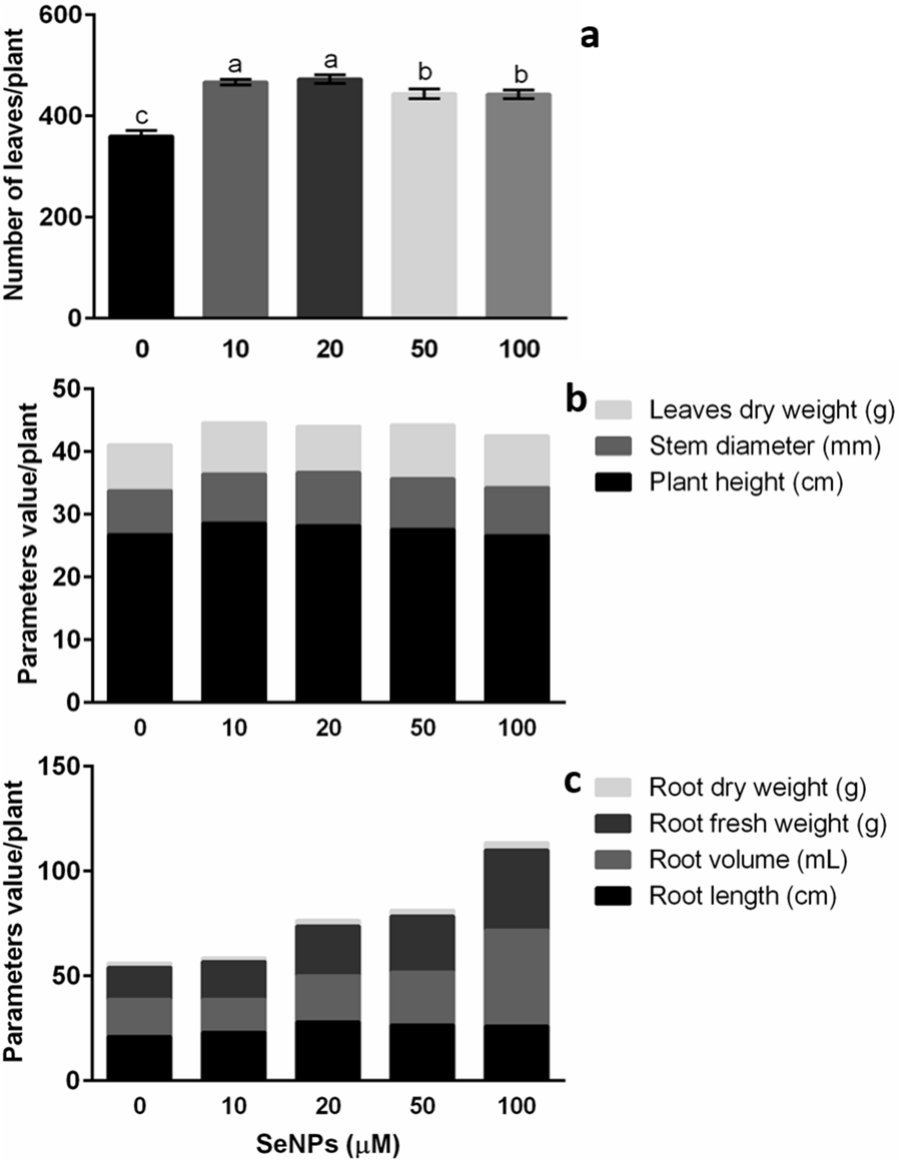
Biostimulant effect of SeNPs on vinca under greenhouse conditions. **a** Number of leaves. **b** Plant height, stem diameter, and leaves dry weight. **c** Root length, root volume, root fresh weight, and root dry weight. Average values ± DE. Different letters in each section denote statistically significant differences according to Duncan's test (α = 0.05), p < 0.0001 (the details are provided in Additional file 2)

Regarding leaf number, the exposure of vinca plants to all SeNP treatments (10–100 μM) increased this parameter by 28% concerning the control (Fig. 7a). In plants, leaf development is regulated by different elements such as transcription factors, miRNA, and hormones [5]. Among plant hormones, ethylene (ET) regulates leaf number through leaf abscission. Inhibition of ethylene action has been identified to reduce this effect. In this sense, SeNPs decrease the impact provoked by ET by lowering ABA levels. Furthermore, with the increase in AUX, leaf abscission is retarded by reducing the sensitivity of the cells to ET [13].

Statistical differences were found for the fresh and dry weights of leaves for all treatments with SeNPs. In the first case, spraying SeNPs produced increases for all treatments, 1.2-fold on average, compared with the control (Table 2). Similar behaviors were observed under in vitro conditions in chili bell pepper seeds, as the SeNP concentrations (0.5–1.0 mg/L) favored growth and increased leaf fresh weight by 65.5% [63].

**Fig. 8:**
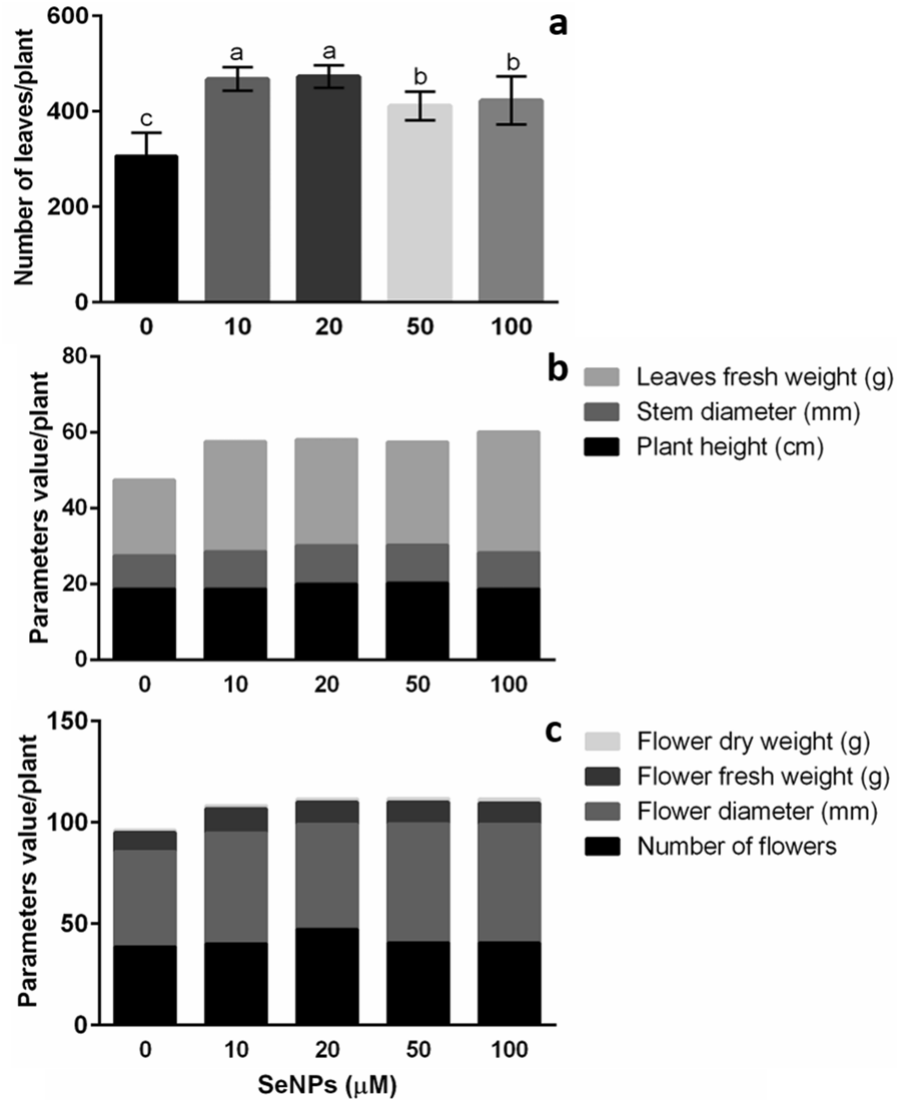
Biostimulant effect of SeNPs on calendula growth under greenhouse conditions. **a** Number of leaves. **b** Plant height, stem diameter, and leaves fresh weight. **c** Number of flowers, diameter of flowers, fresh weight of flowers, and dry weight of flowers. Average values ± DE. Different letters in each section denote significant differences according to Duncan's test (α = 0.05), p < 0.0001 (the details are provided in Additional file 2)

For dry leaf weight, the 50 μM dose increased this value by 16% in untreated plants (Fig. 8b). In another study, the application of SeNPs (0–25 µM) to cowpea (*V. unguiculata*) plants significantly increased growth parameters such as the number, fresh weight and dry weight of leaves [13]. In general, leaf weight is related to leaf size. At this point, hormonal control regulates leaf size and growth. Among the hormones involved in these processes, GA and brassinosteroids (BRs) are distinguished due to favor leaf growth through cell proliferation and expansion [5]. GA increases cell proliferation by regulating cell cycle inhibitors such as KIP-RELATED PROTIEN2 (KRP2), whereas overexpression of the *DWARF4* gene produces larger leaves [63]. Therefore, the application of SeNPs could influence the production of this type of hormone.

The number of branches increased with all SeNP treatments. Within the doses evaluated, 20 μM presented a more significant biostimulant effect, increasing this value by 26% in plants without SeNPs (Table 2). The stem diameter measurement was higher with all SeNP treatments. From this result, 20 μM was established as the best concentration with an increase of 21% compared with the control (Fig. 8b). On the other hand, stem fresh and dry weight followed this same trend for all SeNP treatments evaluated. For fresh biomass, all treatments were significantly superior to the control and equal to each other, obtaining 18% more stem fresh weight on average (Table 2).

In the same way, dry stem weight followed a similar behavior as fresh stem weight. At this point, all concentrations of SeNPs increased by 7% over the weighted average compared with the control (Table 2). Based on these results, the effect produced by SeNPs on plants depends on the dose used. Although, other parameters, such as the size and surface composition of NPs, are also critical points for plant biostimulation [30]. At the same time, several investigations have established that the biostimulant effect is related to primary metabolism. In this regard, low doses have been shown to induce the enzyme nitrate reductase activity (related to primary metabolism). In contrast, high concentrations may cause toxicity by disrupting nitrogen assimilation [1]. In addition, increased uptake of Mo and Fe (cofactors of nitrate reductase) has been reported using SeNPs [30]. This results in higher amino acid and protein contents, leading to biostimulation of plant growth. In addition, the biostimulant effect of SeNPs depends on the surface composition of the NPs and their interaction with other plant cellular components, such as organelles, internal membranes, and cytoplasmic proteins. The application method of SeNPs also has a significant influence on their biostimulant action. For example, foliar spraying decreases the toxic effect of SeNPs on plants due to interactions with some leaf structures, such as cuticles [31]. The application of SeNPs to ornamental flowering plants has been little studied.

The biostimulant effect of SeNPs on flowers greatly affected different parameters where changes such as flower size were observed. Also, the treatments with SeNPs improved the number of flowers at all concentrations used. In this case, no significant differences were observed between the doses of SeNPs, with an average of 13 flowers above those obtained by the control (Table 2). Similarly, Pandey et al. [43] found a higher production of vinca flowers using carbon nanotubes and graphene. Whereas, Salachna et al. [52] reported the ability of SeNPs to induce early flower development and significantly stimulate flower production in chrysanthemum plants. Similar results have been obtained using AgNPs, which produced more flowers in lily plants under treatments with 50–100 mg/L [55].

The fresh weight of vinca flowers increased by 70% with a concentration of 50 μM concerning the control (Table 2). On the other hand, the dry weight of flowers presented a trend similar to that mentioned in the previous parameter. For the best concentration, 50 μM provided a 50% increase in yield to the control (Table 2). For the specific case of SeNPs, the biostimulant effect on flower production could occur because SeNPs increase the levels of transcription factors such as bZIP and WRKY1 (involved in tissue differentiation, organ development, and flowering) [18, 63].

In addition, the effect of SeNPs on the root growth of vinca plants cultivated in greenhouses was evaluated. For this part of the plant, changes in length, root volume, fresh weight, and dry weight were observed (Table 2). Root length was more significant with the application of doses of 20, 50, and 100 µM. These treatments were statistically equal among them, showing on average 1.2 times greater length than the control (Fig. 8c). According to what was reported by Zahedi et al. [75], this behavior may be due to the changes produced by SeNPs in the biosynthesis of AUX, a type of phytohormone capable of stimulating root development, as they indicated that the application of SeNPs (20 mg/L) increased the levels of this phytohormone by 49.3%, thus improving root biomass and the absorption of water and nutrients.

In this work, root volume showed an upward trend as the concentration of SeNPs increased. Likewise, 100 μM was the best treatment, presenting a 157.0% increase concerning untreated plants (Fig. 8c, Additional file 2: Table S1). This suggests an increase in cell proliferation and differentiation. In the leaves of tomato plants under temperature stress, applying SeNPs (8 µM) improved root volume by 60.0% and decreased by 62.5% when SeNPs were not used at low temperatures [21].

The root fresh and dry weights increased with the 20 μM treatment. For fresh and dry biomass, 100 μM was the best treatment with increases of 146.0% (Fig. 8c) and 81.0% (Additional file 2: Table S1) compared with the control, respectively. This result agrees with that obtained by Sheikhalipour et al. [61], who achieved a 3.0% improvement in root dry weight of bitter melon (*Momordica charantia*) plants with the application of 20 mg/L SeNPs.

The chlorophyll *a* content increased with the 50 µM treatment, and this value was 41.0% higher than that of the control. The rest of the treatments were statistically equal to the control (Table 2). Chlorophylls are crucial photosynthetic pigments for the plant that broadly establishes photosynthetic capacity and, therefore, plant development. Certain factors can influence chlorophyll levels. From the perspective of Se, D'Amato et al. [11] noted that an increase in the content of these pigments with low doses of Se is related to a positive effect of this element on chlorophylls biosynthesis by enhancing the flow of electrons in respiration and protecting chloroplast enzymes.

In the case of chlorophyll *b*, this parameter was improved with concentrations of 50 and 100 µM by 32.0 and 6.0%, respectively, for each case concerning the control (Table 2). On the other hand, the total chlorophyll content coincided with the result obtained with chlorophyll *a*. The 50 µM treatment was the best concentration evaluated, with an increase of 32% over the control (Table 2). In contrast, Haghighi et al. [21] showed that, in tomato leaves, SeNPs (1 μM) increased the chlorophyll content by 27.5%, while the use of another Se source, Na_2_SeO_4_, at a higher concentration (2.5 μM) improved the chlorophylls content by 19.2% under low temperature stress. These results highlight the changes that can be found using SeNPs in different cultures. In the case of vinca plants, a higher concentration (50 μM) was required to obtain results comparable with those of tomato plants (1 μM).

The carotenoids content increased on average by 28.0% with all SeNP treatments used concerning the control (Table 2). Improved plant productivity could be due to maintaining cellular ionic and osmotic balance. These processes can optimize photosynthesis by increasing the amount of photosynthetic pigments and decreasing ROS levels [34, 42]. At the same time, carotenoids are crucial in protecting the photosynthetic reaction center, as they act as antioxidants and oxygen scavengers. They are also involved in light protection during photosynthesis, in membrane stability, and in preventing lipid peroxidation, especially under abiotic stress conditions [34]. Therefore, the increase in this type of photosynthetic pigment could be related to the reduction in oxidative damage by enhancing antioxidant capacity [66]. In addition, SeNPs can increase the levels of N and Mg (structural components of chlorophylls), which could benefit the increased accumulation of photosynthetic pigments, leading to a higher photosynthetic rate [13].

The photosynthetic efficiency of PSII showed no variation for any of the SeNP treatments to the control (Table 2). In contrast, reports on the microalga *Chlorella vulgaris* established that SeNPs (0.4–4 mg/L) promoted the photochemical efficiency of photosystem II [42]. It is suggested that SeNPs could be involved in improving photosynthetic efficiency due to an increase in Rubisco activity as part of the Calvin and Benson cycle and by enhancing electron flow between photosystem II and photosystem I in the Hill reaction [24].

### Effect of SeNPs on the growth of calendula plants

According to a search in different databases, this would be the first study evaluating the biostimulant effect of SeNPs on calendula plants. As part of the biostimulant activity of the NPs, significant changes were found in plant growth in terms of plant height, the number of leaves and flowers, as well as stem diameter (Additional file 1: Fig. S2).

First, plant height increased 1.1-fold on average with the 20 and 50 μM concentrations compared with plants without SeNP treatment (Table 3). Additionally, the biostimulant effect of SeNPs was reflected in the number of leaves; in this variable, the concentrations of 10 and 20 μM showed the best results, with significant increases of 1.5 times for both cases, concerning the control (Fig. 8a). The fresh and dry weights of calendula leaves were also favored with the application of SeNPs. For the former, all treatments were significantly superior to the control. In addition, 100 μM was the best of the treatments, increasing the fresh weight of leaves by 59% (Fig. 8b, Additional file 2: Table S2).

**Table 3:** Biostimulant activity of SeNPs on calendula plants development and photosynthetic pigment content

SeNPs (µM)	RM	DWL (g)	FWS (g)	DWS (g)	RL (cm)	RV (mL)	FWR (g)	DWR (g)	Chl*a* (µg g^−1^ FW)	Chl*b* (µg g^−1^ FW)	Chl*t* µg g^−1^ FW)	CR (ng g^−1^ FW)	PE
Control	9.8 ± 0.3a	2.9 ± 0.1b	22.8 ± 0.6b	6.3 ± 0.2ab	18.2 ± 1.3b	20.5 ± 1.6c	20.6 ± 1.4b	2.2 ± 0.1b	1.1 ± 0.1b	0.4 ± 0.1b	1.4 ± 0.1b	0.07 ± 0.1b	0.55 ± 0.1a
10 µM	10.5 ± 0.3a	3.7 ± 0.1a	26.6 ± 0.7a	6.5 ± 0.2ab	23.6 ± 1.3a	33.7 ± 2.3a	30.8 ± 2.8a	2.7 ± 0.2ab	1.0 ± 0.2b	0.4 ± 0.1b	1.4 ± 0.3b	0.07 ± 0.1b	0.56 ± 0.1a
20 µM	10.8 ± 0.7a	3.6 ± 0.1a	29.0 ± 1.3a	7.0 ± 0.3a	21.2 ± 1.4ab	29.3 ± 0.4b	31.6 ± 0.8a	3.2 ± 0.1a	1.5 ± 0.1ab	0.6 ± 0.1ab	2.1 ± 0.2ab	0.11 ± 0.1a	0.53 ± 0.1a
50 µM	10.0 ± 0.4a	3.5 ± 0.1a	26.3 ± 0.7a	6.1 ± 0.2ab	20.5 ± 1.9ab	30.0 ± 0.1b	29.9 ± 1.3a	3.0 ± 0.1a	1.8 ± 0.1a	0.7 ± 0.1a	2.5 ± 0.2a	0.10 ± 0.1ab	0.54 ± 0.1a
100 µM	10.1 ± 0.5a	3.9 ± 0.1a	25.8 ± 1.3ab	5.8 ± 0.3b	17.1 ± 0.5b	30.4 ± 0.4b	30.1 ± 0.8a	2.8 ± 0.1a	1.4 ± 0.1ab	0.6 ± 0.1ab	2.0 ± 0.1ab	0.08 ± 0.1ab	0.52 ± 0.1a

Mean values ± SE. Different letters in each column denote statistically significant differences according to Duncan's test (α = 0.05), p < 0.0001

*RM* Ramifications, *FWS* Fresh weight stem, *DWS* Dry weight stem, *RL* Root length, *RV* Root volume, *FWR* Fresh weight root, *DWR* Dry weight root, *Chl a* Chlorophyll a, *Chl b* Chlorophyll b, *Chl t* Total chlorophyll, *CR* Carotenoids, *PE* Photosynthetic efficiency

On the other hand, leaf yield, determined as dry weight, was improved with all SeNPs treatments, obtaining an average increase of 25% over the control (Table 3). According to Juárez-Maldonado et al. [30], biostimulation of SeNPs could occur in two stages. The first phase consists of the interactions of surface charges (physicochemical nature) between the NPs and the plant. In contrast, the second phase arises from biochemical stimuli originating from the entry of NPs into the plant cells or due to the release of the chemical elements in the NPs.

For the number of branches, no significant changes were found in the control (Table 3). This agrees with the research of Srivastava et al. [64], where applying AuNPs (5–20 mg/L) to calendula plants did not produce significant changes in branching. In contrast, Tripathi et al. [67] found an increase in the number of branches with carbon nanotubes (6.0 mg/mL) in chickpea (*Cicer arietinum*) plants. Plant biostimulation with the use of NPs occurs in various concentrations that depend on the particular type of NPs and their characteristics.

Regarding fresh stem weight, SeNPs increased the fresh biomass by an average of 1.1-fold with the 10, 20, and 50 μM treatments. There were no significant variations in dry stem weight between plants grown with or without SeNPs (Table 3). Stem diameter increased by 17% with the 20 μM treatment to the control (Fig. 8b). The increase in biomass could be related to improved photosynthesis caused by increased levels of photosynthetic pigments through the application of SeNPs. For example, using 100 mg/L AgNPs in lily plants resulted in increased chlorophyll synthesis and more efficient uptake of minerals such as K, Ca, and S, considerably improving plant growth and biomass production [55].

In the case of flowers, SeNPs produced changes in flower size, number of flowers, and fresh and dry weight (Additional file 2: Table S2). The number of flowers increased by 22% with the 20 μM concentration compared with the control, and it was the treatment with the most significant changes. For the rest of the treatments, the data obtained were superior to the control (Fig. 8). In this research, the highest concentration (100 µM) did not induce any signs of phytotoxicity in vinca and calendula plants. This contrasts with the data obtained in chicory (*Cichoriumintybus*) plants as the highest concentration evaluated (40 mg/L) was associated with a risk of cytotoxicity [1]. In general, this could be due to the fact that, in the research with chicory plants, the synthesis of SeNPs was performed by chemical methods. Green methodologies for obtaining NPs offer less toxicity to plants than NPs obtained by chemical methods. For example, the effect of BioSeNPs and CSeNPs on wheat plants was evaluated. A better result was obtained in plants treated with BioSeNPs, which reduced the incidence of crown and root rot disease by 75%. In addition, plant growth, grain quantity, and quality were improved compared with CSeNPs [14].

In this work, when evaluating the effect on flower diameter, an increasing trend was observed with increasing concentrations of SeNPs. In this case, 50 and 100 μM were the best treatments, with an average increase of 24% concerning the control (Fig. 8c). Additionally, the fresh weight of calendula flowers increased with all SeNP treatments; within these concentrations, 100 μM produced a higher yield (50%) to the control (Fig. 8c). Similarly, dry flower weight maintained the same behavior shown with SeNPs for fresh flower weight. In this case, 10 μM proved to be the best concentration of SeNPs, with an increase of 27% to the control (Fig. 8c). In another investigation with calendula plants, a higher flower dry weight yield was reported with titanium dioxide NPs (200 mg/L) [34].

The biostimulant effect of SeNPs on calendula roots was determined with parameters such as volume, length, and fresh and dry weight. Root length was significantly increased only with 10 μM, achieving an increase of 29% concerning the control. The rest of the treatments were statistically equal to the control (Table 3). Regarding root volume, the 10 μM treatment showed the best results, increasing by 64% compared with the result obtained with the control (Table 3). Root fresh weight increased 48% on average compared with the control. For root dry weight, treatments with 20, 50, and 100 μM improved this parameter by 33% on average compared with the control (Table 3). With the data obtained for these parameters, a comparison with the inorganic form of Na_2_SeO_3_ can be made. For example, in the research of Hernández-Díaz et al. [24], using Na_2_SeO_3_ (5–20 µM) on calendula plants did not show significant differences in root length among all treatments evaluated to the control. This could be mainly because SeNPs present a high bioavailability and lower toxicity than the inorganic (Se^2−^, SeO_4_^2−^, and SeO_3_^2−^) and organic (SeMet and SeCys) forms of Se [18].

Regarding chlorophyll content, the treatments with SeNPs showed significant changes. The chlorophyll *a* content increased by 63% compared with the control when the 50 µM concentration was applied (Table 3). The rest of the treatments were statistically similar to the control. The content of chlorophyll *b* and total chlorophylls increased by 75% and 79%, respectively, similar to the trend found in chlorophyll *a*, and the best results were obtained with 50 µM SeNPs (Table 3). In sorghum plants subjected to heat stress, spraying SeNPs (10 mg/L) increased the chlorophyll content, stomatal conductance, and photosynthetic rate. In addition, the damage to the thylakoid membrane decreased by 18% compared with plants without SeNPs, indicating that SeNPs could increase the content of photosynthetic pigments and the protection of the thylakoid membrane by restoring the structure of chloroplasts subjected to high-temperature damage [10]. Therefore, SeNPs could increase the cellular metabolic rate and delay the senescence of chloroplasts, destroying chlorophylls and increasing the biosynthesis of photosynthetic pigments [13].

On the other hand, the carotenoid content increased by 57% with the application of 20 μM compared with the control. The rest of the treatments with SeNPs were statistically similar to untreated plants (Table 3). Finally, in calendula plants, no significant differences were found in the photosynthetic efficiency of PSII in leaves treated with SeNPs (Table 3). Contrary to these results, an adverse effect of ZnNPs on photosystem II efficiency was reported in *Lemna minor* plants as a function of medium pH [66].

## Conclusion

The synthesized SeNPs presented a spherical shape in higher proportion with average sizes of 40–60 nm and prolonged stability of up to 6 months associated with the stabilizing components of the *A. glaucum* extract (CHE1). SeNPs exhibited antibacterial activity comparable to that obtained with CIP antibiotics against the foodborne pathogens *S. marcescens*, *A. faecalis*, and *E. cloacae*. In addition, high in vitro antioxidant activity was found for the CHE1 extract and SeNPs under all three techniques analyzed. Foliar application of 10 µM SeNPs resulted in the best vegetative development of vinca and calendula plants. Meanwhile, concentrations of 50 and 100 µM SeNPs improved parameters related to flower production and photosynthetic pigment content. The biostimulant effects of SeNPs were closely related to the concentration, and no signs of phytotoxicity were observed.

Furthermore, research on the potential use of SeNPs to control the development of disease symptoms caused by phytopathogenic microorganisms in plant experiments is needed. In addition, the effect of SeNPs on the control of oxidative stress in ornamental plants and crops should be investigated in order to increase production yields and improve the quality of edible plant parts enriched with Se, while taking care of the environment and sustainable resource management.

### Supplementary Information

**Additional file 1: Fig. S1.** Biostimulant activity of SeNPs on vinca plants under greenhouse conditions. **Fig. S2.** Biostimulant activity of SeNPs on growth of calendula plants and flowers under greenhouse.

**Additional file 2: Table S1.** Biostimulant effect of SeNPs on shoot and root growth of vinca plants. **Table S2.** Effect of SeNPs on plant shoot growth and calendula flower production.

## Data Availability

All data generated in this study were analyzed and presented in the body of this article and its supplementary files.
