# Supplementary material for: Selenium nanoparticles based on Amphipterygium glaucum extract with antibacterial, antioxidant, and plant biostimulant properties
Source: J Nanobiotechnology. 2023 Aug 3;21:252. doi: 10.1186/s12951-023-02027-6 (PMC10399041; doi:10.1186/s12951-023-02027-6)
Supplement: Supplementary file 1 — Additional file 1: Fig. S1. Biostimulant activity of SeNPs on vinca plants under greenhouse conditions. Fig. S2. Biostimulant activity of SeNPs on growth of calendula plants and flowers under greenhouse. [file 12951_2023_2027_MOESM1_ESM.docx]

**Additional Information 1**

**Selenium nanoparticles based on *Amphipterygium glaucum* extract with antibacterial, antioxidant, and plant biostimulant properties**

Jorge J.O. Garza-García^a‡^, José A. Hernández-Díaz^a‡^, Janet M. León-Morales^b^*, Gilberto Velázquez-Juárez^c^, Adalberto Zamudio-Ojeda^c^, Jenny Arratia-Quijada^d^*, Oscar K. Reyes-Maldonado^c^, Julio C. López-Velázquez^a^, Soledad García-Morales^e^*

^a^Centro de Investigación y Asistencia en Tecnología y Diseño del Estado de Jalisco, Plant Biotechnology. Camino Arenero 1227, Zapopan 45019, Mexico

^b^Coordinación Académica Región Altiplano Oeste, Universidad Autónoma de San Luis Potosí. Carretera Salinas-Santo Domingo 200, Salinas de Hidalgo 78600, Mexico

^c^Centro Universitario de Ciencias Exactas e Ingenierías, Universidad de Guadalajara, Boulevard Gral. Marcelino García Barragán 1421, Guadalajara 44430, Mexico

^d^Departamento de Ciencias Biomédicas, Centro Universitario de Tonalá, Universidad de Guadalajara, Av. Nuevo Periférico Oriente 555, Tonalá 45425, Mexico

^e^CONACYT-Centro de Investigación y Asistencia en Tecnología y Diseño del Estado de Jalisco, Plant Biotechnology. Camino Arenero 1227, Zapopan 45019, Mexico


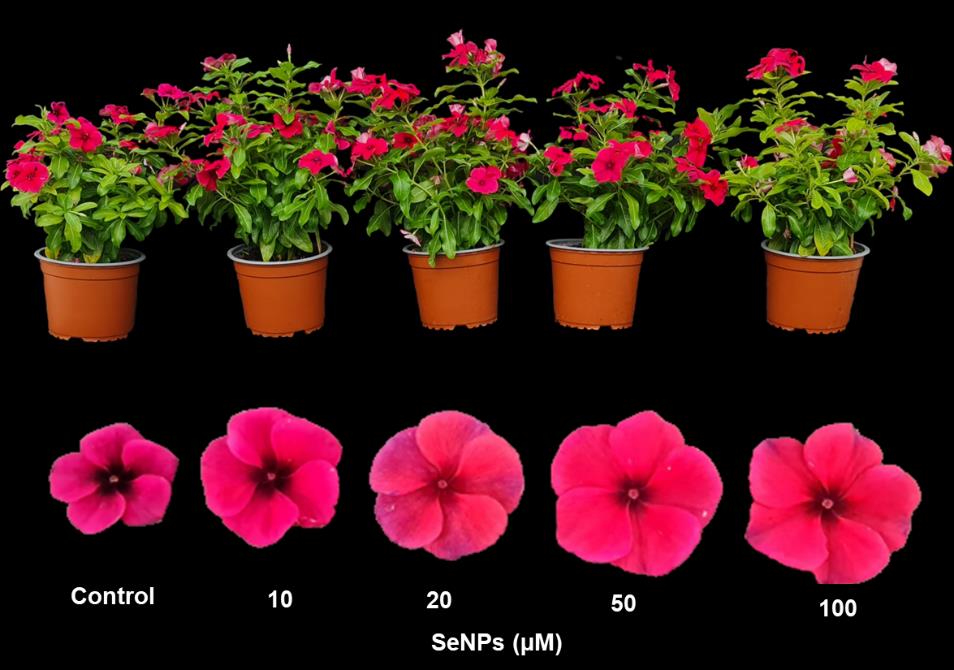


**Fig. S1** Biostimulant activity of SeNPs on vinca plants under greenhouse conditions.


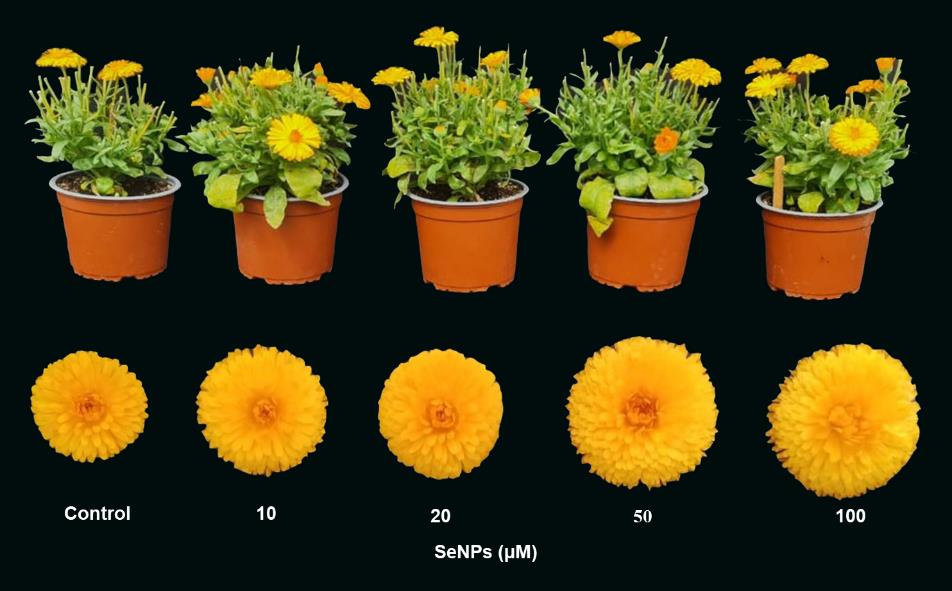


**Fig. S2** Biostimulant activity of SeNPs on growth of calendula plants and flowers under greenhouse.
