# Supplementary material for: Selenium nanoparticles based on Amphipterygium glaucum extract with antibacterial, antioxidant, and plant biostimulant properties
Source: J Nanobiotechnology. 2023 Aug 3;21:252. doi: 10.1186/s12951-023-02027-6 (PMC10399041; doi:10.1186/s12951-023-02027-6)
Supplement: Supplementary file 2 — Additional file 2: Table S1. Biostimulant effect of SeNPs on shoot and root growth of vinca plants. Table S2. Effect of SeNPs on plant shoot growth and calendula flower production. [file 12951_2023_2027_MOESM2_ESM.docx]

**Additional Information 2**

**Selenium nanoparticles based on *Amphipterygium glaucum* extract with antibacterial, antioxidant, and plant biostimulant properties**

Jorge J.O. Garza-García^a‡^, José A. Hernández-Díaz^a‡^, Janet M. León-Morales^b^*, Gilberto Velázquez-Juárez^c^, Adalberto Zamudio-Ojeda^c^, Jenny Arratia-Quijada^d^*, Oscar K. Reyes-Maldonado^c^, Julio C. López-Velázquez^a^, Soledad García-Morales^e^*

^a^Centro de Investigación y Asistencia en Tecnología y Diseño del Estado de Jalisco, Plant Biotechnology. Camino Arenero 1227, Zapopan 45019, Mexico

^b^Coordinación Académica Región Altiplano Oeste, Universidad Autónoma de San Luis Potosí. Carretera Salinas-Santo Domingo 200, Salinas de Hidalgo 78600, Mexico

^c^Centro Universitario de Ciencias Exactas e Ingenierías, Universidad de Guadalajara, Boulevard Gral. Marcelino García Barragán 1421, Guadalajara 44430, Mexico

^d^Departamento de Ciencias Biomédicas, Centro Universitario de Tonalá, Universidad de Guadalajara, Av. Nuevo Periférico Oriente 555, Tonalá 45425, Mexico

^e^CONACYT-Centro de Investigación y Asistencia en Tecnología y Diseño del Estado de Jalisco, Plant Biotechnology. Camino Arenero 1227, Zapopan 45019, Mexico

Table S1. Biostimulant effect of SeNPs on shoot and root growth of vinca plants.

| **SeNPs (µM)** | **Plant height (cm)** | **Stem diameter (mm)** | **Leaves dry weight (g)** | **Root length (cm)** | **Root volume (mL)** | **Root fresh weight (g)** | **Root dry weight (g)** |
| --- | --- | --- | --- | --- | --- | --- | --- |
| 0 | 26.8±1.0 cd | 7.00±0.27 c | 7.321±0.42 c | 21.0±1.4 c | 17.7±2.5 cd | 15.5±2.0 c | 1.978±0.19 c |
| 10 | 28.6±0.9a | 7.85±0.17 b | 8.164±0.42 ab | 23.2±2.7 bc | 15.8±1.0 d | 18.0±1.0 c | 1.817±0.05 c |
| 20 | 28.2±0.5 ab | 8.49±0.37 a | 7.391±0.43 bc | 28.0±1.8 a | 22.1±2.18 bc | 23.7±2.4 b | 2.759±0.45 b |
| 50 | 27.6±0.5 bc | 8.10±0.24 ab | 8.564±0.23 a | 26.5±2.2 ab | 25.4±2.8 bc | 26.7±2.8 b | 2.811±0.21 b |
| 100 | 26.6±0.7 d | 7.72±0.18 b | 8.184±0.67 ab | 26.1±0.9 ab | 45.8±3.2 a | 38.1±3.5 a | 3.584±0.31 a |

Mean values ± DE. Different letters in each column denote statistically significant differences according to Duncan's test (α = 0.05), p <0.0001.

Table S2. Effect of SeNPs on plant shoot growth and calendula flower production.

| **SeNPs (µM)** | **Plant height (cm)** | **Stem diameter (mm)** | **Leaves fresh weight (g)** | **Number of flowers** | **Flower diameter (mm)** | **Flowers fresh weight (g)** | **Flowers dry weight (g)** |
| --- | --- | --- | --- | --- | --- | --- | --- |
| 0 | 18.8±0.3 b | 8.77±0.20 c | 19.925±1.61 c | 38.6±2.1 c | 47.07±1.55 c | 9.37±0.36 c | 1.318±0.05 c |
| 10 | 18.8±0.2 b | 9.86±0.40 ab | 28.986±1.99 ab | 40.0±2.5 b | 54.88±0.57 b | 11.94±0.28 a | 1.652±0.14 b |
| 20 | 20.0±0.9 a | 10.25±0.23 a | 28.010±2.00 ab | 47.3±3.3 a | 51.71±0.79 b | 11.08±0.74 b | 1.655±0.07 b |
| 50 | 20.3±0.5 a | 10.02±0.20 ab | 27.173±1.94 b | 40.7±2.3 b | 58.70±0.60 a | 10.70±0.23 b | 1.811±0.04 ab |
| 100 | 18.8±0.5 b | 9.58±0.12 b | 31.803±1.13 a | 40.6±3.0 b | 58.557±0.93 a | 10.46±0.75 b | 1.979±0.09 a |
